# Impacts of Reduced Nitrate Supply on Nitrogen Metabolism, Photosynthetic Light-Use Efficiency, and Nutritional Values of Edible *Mesembryanthemum crystallinum*

**DOI:** 10.3389/fpls.2021.686910

**Published:** 2021-06-04

**Authors:** Jie He, Lin Qin

**Affiliations:** National Institute of Education, Nanyang Technological University, Singapore, Singapore

**Keywords:** leaf growth, nitrate, nitrate reductase activity, photosynthetic performance, phytochemicals

## Abstract

*Mesembryanthemum crystallinum* (common ice plant), as a nutritious ready-to-eat salad in Singapore, has become popular in recent years. However, basic data about the impacts of NO_3_^–^ supply on its NO_3_^–^ accumulation and nutritional quality are lacking. In this study, all plants were first grown indoor hydroponically in 10% artificial seawater (ASW) with modified full-strength Netherlands Standard Composition nutrient solution for 11 days, before transferring them to different reduced NO_3_^–^ solutions. All plants grew well and healthy after 7 days of treatment. However, plants grown with 3/4 N and 1/2 N were bigger with higher shoot and root fresh weight (FW), greater leaf number, and total leaf area (TLA) than those grown with full nitrogen (N), 1/4 N, and 0 N. *Mesembryanthemum crystallinum* grown with full N, 3/4 N, and 1/4 N had similar specific leaf area (SLA), while 0 N plants had significantly lower SLA. All plants had similar leaf succulence (LS). However, leaf water content (LWC) was lower, while leaf dry matter accumulation (LDMC) was higher in 0 N plants after 7 days of treatment. Compared with plants grown with full N, shoot NO_3_^–^ concentrations in 3/4 N, 1/2 N, and 1/4 N plants were constant or slightly increased during the treatments. For 0 N plants, shoot NO_3_^–^ concentration decreased significantly during the treatment compared with other plants. Shoot NO_3_^–^ accumulation was associated with nitrate reductase activity (NRA). For instance, after 7 days of treatment, shoot NO_3_^–^ concentration and NRA on a FW basis in 0 N plants were, respectively, 45 and 31% of full N plants. After transferring full N to 0 N for 7 days, all *M. crystallinum* had higher chlorophyll (Chl) content coupled with higher electron transport rate (ETR) and higher effective quantum yield of PSII, while full N plants had higher non-photochemical quenching (NPQ). The 0N plants had much higher concentrations of proline, total soluble sugar (TSS), and total ascorbic acid (ASC) than other plants. In conclusion, totally withdrawing NO_3_^–^ from the growth media prior to harvest could be one of the strategies to reduce shoot NO_3_^–^ concentration. Reduced NO_3_^–^ supply further enhanced nutritional values as concentrations of proline, TSS, and ASC were enhanced markedly in *M. crystallinum* plants after transferring them from full N to 0 N.

## Introduction

As Singapore is land-scarce and also faces water shortage, the exploitation of growing halophytic plants as edible vegetables could become an interesting method of enhancing food security. *Mesembryanthemum crystallinum* (common ice plant) is an edible green, whose use in gourmet cuisine has been recommended (Ventura et al., 2015). Due to its special taste and texture, the easy ice plant salad with soy sesame dressing has become popular in recent years in Singapore.

Our recent study showed that *M. crystallinum* does not necessarily need salinity to grow (He et al., 2017, 2020), but it requires saline conditions to achieve optimal growth (He and Qin, 2020). Our studies also showed that *M. crystallinum* grown indoors was affected by LED spectral quality (He et al., 2017), drought stress (He et al., 2020) when plants were grown with freshwater, and NaCl salinity (He and Qin, 2020). A combination of NaCl salinity with LED spectral quality also affected the growth of *M. crystallinum* (He et al., 2021a). These studies showed that salinity, water supply, and light quality affected photosynthetic performance and nutritional quality of *M. crystallinum* grown indoors under LED lighting. However, *M. crystallinum* plants grown under drought (He et al., 2020) and salt stress (unpublished) conditions had adequate shoot total reduced nitrogen (N), which was greater than 2%. According to Epstein (1999), the adequate tissue level of N required by plants is around 1.5%. NO_3_^–^ is the major N source available in the nutrient solution that most frequently limits plant growth (Marschner, 1995). However, according to our previous studies, N deficiency did not occur in *M. crystallinum* when they were grown under drought (He et al., 2020) or salinity conditions (unpublished). We often question whether *M. crystallinum* plants receive too much N and accumulate excessive NO_3_^–^ in the edible shoots when grown with a *soilless medium* under low light conditions.

Vegetables supply more than 70% of the total NO_3_^–^ intake by human beings (Dich et al., 1996). With regard to the NO_3_^–^ content in lettuce, the maximum limits prescribed by European Union for lettuce produced in open field are 2,500–4,000 mg kg^−1^ fresh weight (FW) and for lettuce produced in the greenhouse are 3,500–4,500 mg kg^−1^ grown (Zhong et al., 2002). In the recent study with a nutritious leafy halophyte vegetable, *Portulaca oleracea L* (known as purslane) grown hydroponically, we have found that the NO_3_^–^ concentrations of purslane grown with 0, 100, 200, and 300 mM NaCl were 1,868, 2,081, 1,381, and 1,235 mg kg ^−1^ FW, respectively (He et al., 2021b). However, for *M. crystallinum* plants grown under the same conditions, we have yet to determine the NO_3_^–^ concentrations in their edible shoots.

It has been reported that a high level of NO_3_^–^ accumulation in plants is harmful to both human health (Ishiwata et al., 2002) and plant growth (Reddy and Menary, 1990; Nakaji et al., 2002; Yao et al., 2016; Cun et al., 2021). In the study of the native Australian plant boronia (*Boronia megastigma* Nees), Reddy and Menary (1990) reported that at a low level of NO_3_^–^ supply, NO_3_^–^ was reduced without any detectable accumulation and without nitrate reductase activity (NRA) reaching its maximum capacity. However, when NO_3_^–^ supply is high, the plant cannot reduce it at high rates, leading to the accumulation of large amounts of the toxic NO_3_^–^ ion in the tissues of the leaf. In the study with Japanese red pine (*Pinus densiflora*) seedlings, Nakaji et al. (2002) found that high N application reduced biomass accumulation, which was mainly due to the reduced photosynthetic rate and the declined PSII activity. Excessive N induced the decline in photosynthetic efficiency, which has also been reported by Yao et al. (2016) for cotton (*Gossypium hirsutum*). In the study with Chinese ginseng or notoginseng (*Panax notoginseng*), Cun et al. (2021) found that plants were readily exposed to photooxidation under excessive N stress. Their study showed that the photosynthetic capacity might be primarily inhibited by the inactivated Rubisco in the high N (HN)-grown plants, and HN-induced depression of photoprotection might be caused by the photodamage to the donor side of PSII oxygen-evolving complex. In the study with two lettuce (*Lactuca sativa*) recombinant inbred lines (RILs), He et al. (2014) have previously reported that the shoot and root productivity of both RILs grown under high N (+N, 125% NO_3_^–^) and low N (–N, 50% NO_3_^–^) declined compared with those of full N plants (100% NO_3_^–^). Shoot NO_3_^–^ concentration was highest in high N plants followed by full N plants, and low N plants had the lowest values. High N and full N plants had similar higher shoot maximal NRA than low N plants.

Today, there is a major paradigm shift in how we perceive food, from the traditional concept of carbohydrate, protein, fat, and calories toward critical functional molecules. Phytochemicals such as phenolic compounds and ascorbic acid (ASC) have health-promoting properties (Boestfleisch et al., 2014). To protect against hyperosmotic stress caused by salinity, halophyte species produced osmolytes such as proline and total soluble sugars (TSS) that can be utilized in functional food (Hsouna et al., 2020). It has been reported that the production of phytochemical is influenced by both environmental factors and cultivation methods (Ou et al., 2002). N availability plays a key role in the production of phytochemicals in vegetables, fruit, herbal plants, and medicinal plants (Sorensen, 1993; Le Bot et al., 2001; Khan et al., 2018). For instance, high N supply reduced ASC contents in vegetables without reducing yield (Sorensen, 1993). In the study with three lettuce cultivars, Khan et al. (2018) found that an increase in light intensity combined with a decrease in NO_3_^–^ supply led to the increase in the concentration of nutritional carbohydrates and a gradual decrease in NO_3_^–^ content prior to harvest. Le Bot et al. (2001) demonstrated that N supply positively correlated with fruit yield up to a threshold value; above the threshold value, overfeeding of plants did not increase the yield. Despite the increasing consumption of *M. crystallinum* plants as a read-to-eat salad in Singapore, basic data about the effects of NO_3_^–^ supply on its growth, NO_3_^–^ content, and nutritional quality are still lacking. Therefore, this study aimed to determine the effects of NO_3_^–^ supply on plant growth, N metabolism, photosynthetic light-use efficiency, and phytochemicals in edible *M. crystallinum* grown in the controlled environment.

## Materials and Methods

### Plant Materials and Culture Methods

Seeds of *M. crystallinum* were germinated on a filter paper before being inserted into polyurethane cubes and incubated under a photosynthetic photon flux density (PPFD) of 100 μmol m^−2^ s^−1^ provided by high-pressure sodium lamps for 3 weeks. Seedlings were then transplanted into an indoor hydroponic system with 10% artificial seawater (ASW, Red Sea Salt). A modified full-strength Netherlands Standard Composition nutrient solution (defined as Full N) with 2.2 ± 0.2 mS cm^−1^ conductivity was added to 10% ASW before transplanting. The modified full-strength Netherlands Standard Composition solution had the following composition: N, 13.37 mM; P, 1.07 mM; K, 7.90 mM; Ca, 5.25 mM; Mg, 2.47 mM; S, 3.91 mM; Fe, 0.17 mM; B, 9.71 μM; Mn, 4.33 μM; Zn, 0.21 μM; Cu, 0.24 μM; and Mo, 4.25 μM. The pH of the nutrient solution in 10% ASW was 6.0 ± 0.2. All plants were grown under LED lamps with red/blue (R/B) ratio of 2.2 (WR-16W, Beijing Lighting Valley Technology Co., Ltd., China), and the light spectral distribution is shown in Supplementary Figure S1. All plants were exposed to the same level of PPFD of 240 μmol m^−2^ s^−1^, 12-h photoperiod. The room temperature and relative humidity were 24.5/23°C and 56/82% (day/night), respectively.

### Different NO_3_^–^ Treatments

Around 11 days after cultivating with full N condition described above, *M. crystallinum* plants were divided into five groups, and each of them was supplied with one of the five different NO_3_^–^ concentrations: 100% NO_3_^–^ (13.37 mM, full N), 75% NO_3_^–^ (10.03 mM, 3/4 N), 50% NO_3_^–^ (6.69 mM, 1/2 N), 25% NO_3_^–^ (3.34 mM, 1/4 N), and 0% NO_3_^–^ (0.00 mM, 0 N). Full N plants were supplied with modified full-strength Netherlands Standard Composition described above. Nutrient solution conductivity and pH were maintained at 2.2 ± 0.2 mS cm^−1^ and 6.0 ± 0.2, respectively. For 3/4 N, 1/2 N, 1/4 N, and 0 N treatments, the NO_3_^–^ supplied from the KNO_3_ and Ca(NO_3_)_2_.4H_2_O was reduced accordingly compared to full N nutrient solution, and different amounts of KCl and CaCl_2_.2H_2_O were added, respectively, into the different nutrient solutions to compensate the different amounts of K and Ca. All plants were grown in the same culture room under the same light, temperature, and humidity as described above. Plant samples were harvested for different analyses at 2 and 7 days after different NO_3_^–^ treatments.

### Measurements of Shoot and Root Productivity, Leaf Growth, and Leaf Water Status

After harvest, the total number of leaves was recorded. Shoot and root were separated for fresh weight (FW) measurement. The youngest fully expanded leaves were also weighed separately. Total leaf area (TLA) and the area of the youngest fully expanded leaf were measured using a leaf area meter (WinDIAS3 Image Analysis system, Delta-T, the United Kingdom). Leaves and roots were then dried at 80°C for 4 days, before reweighing them to obtain dry weight (DW). Specific leaf area (SLA) was determined as L_a_/L_DW_, where L_a_ = leaf area (cm^2^) and L_DW_ = leaf dry weight (g; Hunt et al., 2002). Leaf succulence (LS) was estimated as L_FW_/L_a_, where L_FW_ = leaf FW (Agarie et al., 2007). Leaf dry matter content (LDMC) was determined by L_DW_/L_FW_ (Garnier et al., 2001). Leaf water content (LWC) was determined as (L_FW_−L_DW_)/L_FW_.

### Measurements of Chlorophyll Concentration

Leaf disks (1 cm diameter) cut from the youngest fully expanded leaves were weighed and soaked in 5 ml of *N, N*-dimethylformamide (*N,N*-DMF, Sigma Chemical Co.) in darkness for 48 h at 4°C. The absorbance of pigments was measured using a spectrophotometer (UV-2550 Shimadzu, Nagoya, Japan) at 647, 664, and 480 nm. Chlorophyll (Chl) a, Chl b, and total Chl concentrations were calculated as described by Wellburn (1994).

### Measurement of Chl Fluorescence F_v_/F_m_ Ratio

Maximum photochemical efficiency of PS II was estimated in the leaf samples adapted to darkness for 15 min by the F_v_/F_m_ ratio during mid-photoperiod using the plant efficiency analyzer (Hansatech Instruments, the United Kingdom).

### Measurements of Electron Transport Rate, Effective Quantum Yield of PSII (ΔF*/*F_m_'), and Non-photochemical Quenching

After 7 days of NO_3_^–^ treatments, the youngest fully expanded leaves were harvested and electron transport rate (ETR), ΔF*/*F_m_', and non-photochemical quenching (NPQ) were determined at 25°C in the laboratory. Prior to measurements, the leaves were pre-darkened for 15 min. By using the IMAGING PAM MAXI (Walz, Effeltrich, Germany), images of fluorescence emission were digitized within the camera and transmitted *via* an Ethernet interface (GigE Vision®) to a personal computer for storage and analysis. After determining F_v_/F_m_ ratio, rapid light curve measurements in the presence of actinic illuminations were obtained through the application of a series of 10-s light exposures with increasing irradiance from 1 to 1,076 μmol photons m^−2^ s^−1^. Measurements and calculations of ETR, ΔF*/F*_m_', and NPQ were determined according to that demonstrated by He et al. (2011b).

### Measurement of NO_3_^–^ Concentration

NO_3_^–^ concentration was determined using a Flow Injection Analyzer (Model QuikChem 8000, Lachat Instruments Inc., Milwaukee, WI, the United States) according to study by He et al. (2020). Dried tissues of 0.01 g were ground with Mill-Q water and then incubated at 37°C for 2 h. Sample turbidity was removed by filtration through a 0.45-μm pore diameter membrane filter, and the final volume was made to 50 ml prior to analysis. The NO_3_^–^ was determined using the Flow Injection Analyzer by catalytically reducing NO_3_^–^ to NO_2_^–^ by passage of the sample through a copperized cadmium column. The NO_2_^–^ was then determined by diazotizing with sulfanilamide followed by coupling with *N*-(1-naphthyl)ethylenediamine dihydrochloride. The resulting water-soluble dye had a magenta color, which was read at 520 nm.

### Determination of Maximal NRA

Leaf samples harvested from fully expanded young leaves were frozen rapidly in liquid nitrogen after being weighed and stored at −80°C until use. Tissue samples were extracted with the high-pH Tris–HCl buffer (pH 8.5) developed by Kuo et al. (1982). A frozen sample of 1 g was powdered in liquid nitrogen and ground with 4 ml of extraction buffer, with a mortar and pestle in the presence of 0.2 g/g FW insoluble PVP. The extraction buffer consisted of 0.25 M Tris–HCl (pH 8.5), 3 mM dithiothreitol (DTT), 10 μM flavin adenine dinucleotide (FAD), 1 μM sodium molybdate, and 1 mM ethylenediamine tetra-acetic acid (EDTA). The extracts were centrifuged at 18,000 *g* for 20 min at 4°C. NRA was measured immediately in the supernatant.

*In vitro* NADH:NR activity assay was derived from Kaiser and Huber (1997) with modification. The maximum activity of NR was determined by assaying NR with EDTA (15 mM). The total reaction medium was 700 μl, which contained 50 mM Hepes–KOH (pH 7.5), 1 mM DTT, 10 μM FAD, 10 mM KNO_3_, 0.2 mM NADH, NR extraction, and 15 mM EDTA. The reaction was started by adding 100 μl NR extraction. Incubation was performed at 25°C for 20 min, and the reaction was then terminated by the addition of an equal volume (700 μl) of sulfanilamide [1%(w/v) in 3 N HCl] and the naphthylethylene-diamine dihydrochloride (0.02% w/v). After incubation for 30 min at room temperature, the absorbance at 540 nm of all the samples was read. The blank was identical to the samples, but the NR extracts were boiled for 5 min before adding into the reaction mixture. NRA was expressed as μmol nitrite h^−1^ g^−1^ FW.

### Measurements of Leaf Total Soluble Protein

Frozen leaf samples of 1 g were ground in liquid nitrogen. The details of extraction processes were described in He et al. (2017). The amount of leaf total soluble protein was determined using the method demonstrated by Lowry et al. (1951).

### Determination of Total Soluble Sugars

Dry samples of 10 mg were used to extract TSS using hot 80% ethanol. The details of extraction processes were described in the study by He et al. (2020). The concentration of free soluble sugar was determined colorimetrically at 490 nm using a spectrophotometer (UV-2550 Shimadzu, Nagoya, Japan) as demonstrated by Dubois et al. (1956).

### Determination of Proline Concentration

This assay was modified from the protocol by Bates et al. (1973) using frozen leaf tissues of 0.5 g. The details of extraction processes and the measurements of absorbance at 520 nm using a spectrophotometer (UV-2550 Shimadzu, Nagoya, Japan) were described in the study by He et al. (2020).

### Measurement of Ascorbic Acid

Total ASC was assayed from 0.5 g of frozen leaves by the reduction of 2,6-dichlorophenolindophenol (DCPIP) as demonstrated by Leipner et al. (1997) and as modified by He et al. (2020). The ASC concentrations were spectrophotometrically assayed by measuring the absorbance at 524 nm using a spectrophotometer (UV-2550 Shimadzu, Nagoya, Japan). L-ASC was used as a standard.

### Determination of Total Phenolic Compounds

Frozen leaf samples (0.5 g) were ground in liquid nitrogen and 5 ml of 80% methanol (Ragaee et al., 2006). The details of extraction processes and the measurements of absorbance at 765 nm using a spectrophotometer (UV-2550 Shimadzu, Nagoya, Japan) were described in the study by He et al. (2020). Gallic acid (GA) was used as a standard. Total phenolic compounds of the samples were expressed as GA equivalents in micrograms per gram of tissue on a FW basis.

### Statistical Analysis

A one-way ANOVA was used to test for significant differences of different variances across the five NO_3_^–^ treatments. Fisher’s least significant difference (LSD) multiple comparison tests were used to discriminate the means (IBM SPSS Statistics 26).

## Results

### Productivity and Leaf Growth

**Figure 1:**
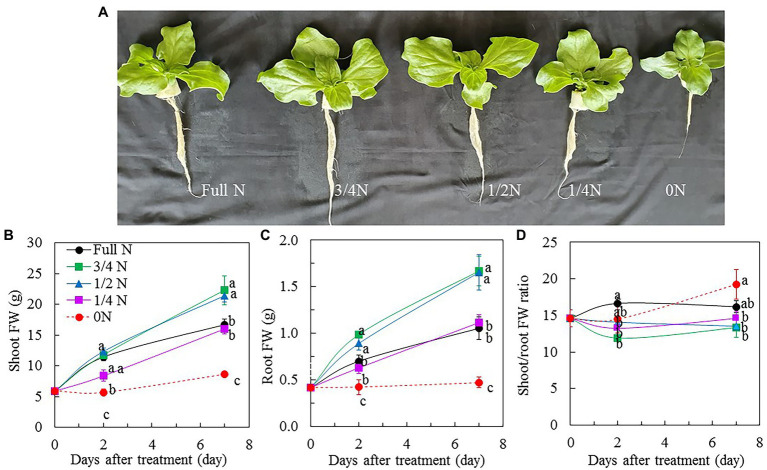
*Mesembryanthemum crystallinum* grown indoor hydroponically for 18 days with different levels of NO_3_^–^ supply **(A)**. Changes in shoot fresh weight (FW; **B**), root FW **(C)**, and shoot/root FW ratio **(D)** of *M. crystallinum* after different NO_3_^–^ treatments. Vertical bars represent the SEs. Means with different letters are statistically different (*p* = 0.05; *n* = 5) as determined by LSD multiple comparison test.

**Figure 2:**
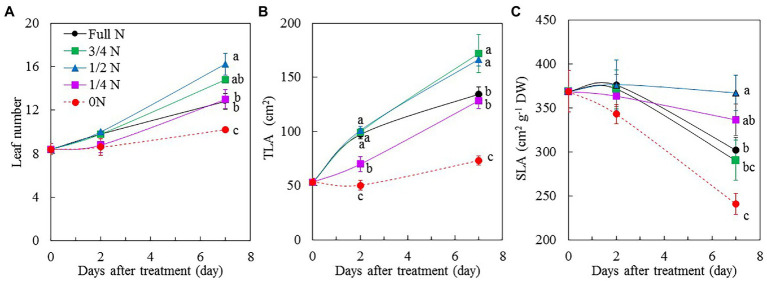
Changes of leaf number **(A)**, total leaf area (TLA; **B**), and specific leaf area (SLA; **C**) of *M. crystallinum* after different NO_3_^–^ treatments. Vertical bars represent the SEs. Means with different letters are statistically different (*p* = 0.05; *n* = 5) as determined by LSD multiple comparison test.

All *M. crystallinum* plants grew well and appeared healthy after transferring them from full N to different reduced NO_3_^–^ nutrient solution for 7 days, although plants grown with 3/4 N and 1/2 N were bigger than those grown with full N, 1/4 N, and 0 N conditions (Figure 1A). The different sizes of *M. crystallinum* plants were supported by their shoot FW (Figure 1B), root FW (Figure 1C), leaf number (Figure 2A), and TLA (Figure 2B). Shoot and root FW of *M. crystallinum* plants transferred from full N to 3/4 N and 1/2 N for 2 days were similar to those of full N plants but significantly higher than those transferred from full N to 1/4 N and 0 N. Although all plants continued to grow, *M. crystallinum* grown with 3/4 N and 1/2 N had the greatest shoot and root FW followed by those grown with full N and 1/4 N, and then those grown with 0 N had the lowest values after 7 days of treatment (Figures 1B,C). For shoot/root FW ratio, *M. crystallinum* which were transferred from full N to 0 N for 7 days had a significantly higher value compared with those transferred to 3/4 N, 1/2 N, and 1/4 N (Figure 1D). Responses of leaf number and TLA to different NO_3_^–^ treatments (Figures 2A,B) were similar to those of shoot and root FW. There were no significant differences in SLA between *M. crystallinum* grown with full N and those transferred from full N to 3/4 N and 1/4 N. However, *M. crystallinum* plants transferred from full N to 1/2 N and to 0 N, respectively, had higher and lower SLA, respectively, than that of full N plants (Figure 2C).

### Leaf Water Status

**Figure 3:**
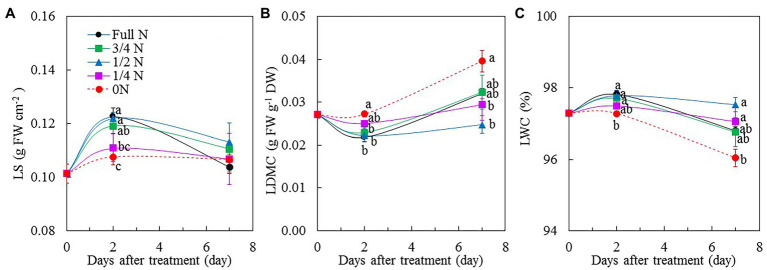
Changes of leaf succulence (LS; **A**), leaf dry matter accumulation (LDMC; **B**), and leaf water content (LWC; **C**) of *M. crystallinum* after different NO_3_^–^ treatments. Vertical bars represent the SEs. Means with different letters are statistically different (*p* = 0.05; *n* = 5) as determined by LSD multiple comparison test.

Around 2 days after transferring *M. crystallinum* plants from full N to 0 N, they had significantly lower LS compared to all other treatments except for 1/4 N plants (Figure 3A). There were no significant differences in LS among all plants after transferring them from full N to different reduced N conditions for 7 days (Figure 3A). Although the trend for LWC was similar to that of LS, the value of LWC for *M. crystallinum* transferred from full N to 0 N for 7 days was significantly lower than those of 1/2 N and 1/4 N plants (Figure 3C). The responses of LDMC to different NO_3_^–^ treatments showed opposite trends compared with those of LS and LWC. There was no significant difference in LDMC between *M. crystallinum* plants transferred from full N to 0 N and to 1/4 N for 2 days, but LDMC in these plants were significantly higher than those of full N, 3/4 N, and 1/2 N plants. After 7 days of different reduced NO_3_^–^ treatments, those with 0 N had the highest LDMC (Figure 3B).

### Shoot NO_3_^–^ Concentration, NRA, and Total Leaf Soluble Protein

**Figure 4:**
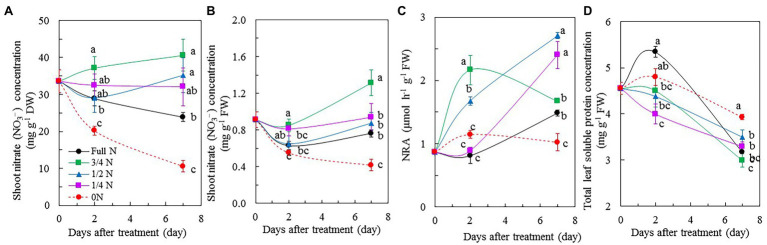
Changes of shoot NO_3_^–^ concentration based on dry weight (DW; **A**), shoot NO_3_^–^ concentration based on FW **(B)**, nitrate reductase activity (NRA; **C**), and total soluble protein **(D)** of *M. crystallinum* after different NO_3_^–^ treatments. Vertical bars represent the SEs. Means with different letters are statistically different (*p* = 0.05; *n* = 5 for Figures 6A,B; *n* = 4 for Figures 6C,D) as determined by LSD multiple comparison test.

Shoot NO_3_^–^ concentration, either on a DW basis (Figure 4A) or a FW basis (Figure 4B), of *M. crystallinum* plants transferred from full N to 0 N decreased during the 7 days of treatment compared to those maintained with full N supply. Shoot NO_3_^–^ concentrations of *M. crystallinum* plants transferred from full N to 3/4 N, 1/2 N, and 1/4 N seemed to have stayed constant or even slightly increased as opposed to decreased during the treatment (Figures 4A,B). After 7 days of treatment, 0 N plants had the lowest shoot NO_3_^–^ concentrations followed by those maintained under full N condition. *Mesembryanthemum crystallinum* plants transferred from full N to 3/4 N and to 1/2 N for 7 days had significantly higher NO_3_^–^ concentrations compared with those supplied with full N on a DW basis (Figure 4A). On a FW basis, only those transferred to 3/4 N had significantly higher NO_3_^–^ concentrations than the rest of plants (Figure 4B). The maximum NRA was low in *M. crystallinum* grown with full N before the treatments. After transferring them from full N to 0 N, the maximum NRA remained at the similar low level. However, those transferred from full N to 3/4 N and 1/2 N had significantly higher maximum NRA compared with other plants after 2 days of treatment. After 7 days of treatment, *M. crystallinum* plants transferred from full N to 1/2 N and 1/4 N had the highest maximal NRA, while those transferred to 3/4 N had a similar value as those grown with full N (Figure 4C). The concentrations of total leaf soluble protein in *M. crystallinum* plants gradually decreased during the reduced NO_3_^–^ treatments except for those of full N or 0 N plants after 2 days of treatment. *Mesembryanthemum crystallinum* plants transferred from full N to 0 N had the highest total leaf soluble protein compared with other plants after 7 days of treatment (Figure 4D).

### Photosynthetic Pigments, F_v_/F_m_ Ratio, ETR, ΔF/F_m_', and NPQ

**Figure 5:**
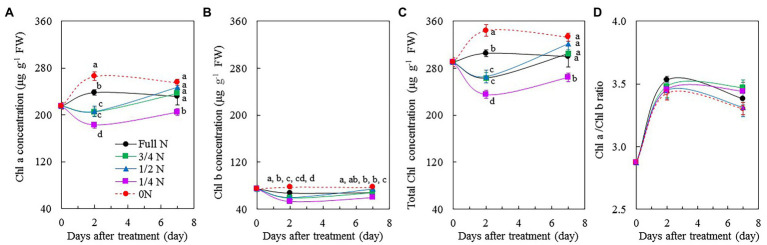
Changes of Chl a concentration **(A)**, Chl b concentration **(B)**, total Chl concentration **(C)**, and Chl a/b ratio **(D)** of *M. crystallinum* after different NO_3_^–^ treatments. Vertical bars represent the SEs. Means with different letters are statistically different (*p* = 0.05; *n* = 5) as determined by LSD multiple comparison test.

Chl a, Chl b, and total Chl concentrations in *M. crystallinum* plants transferred from full N to 0 N were markedly higher than those of other plants (Figures 5A–C) after 2 days of treatment. However, 7 days after treatment, only 1/4 N plants had significantly lower Chl a, Chl b, and total Chl concentrations compared with other plants, which had similar values of these three parameters (Figures 5A–C). All plants had similar Chl a/b ratio before and during the different treatments (Figure 5D).

**Figure 6:**
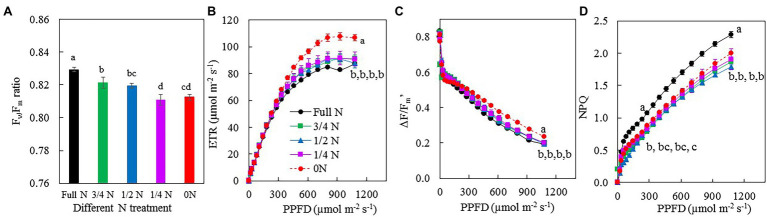
F_v_/F_m_ ratio **(A)**, light-response curves of electron transport rate (ETR; **B**), ΔF/F_m_' **(C)**, and NQP **(D)** of *M. crystallinum* after 7 days of different NO_3_^–^ treatments. Vertical bars represent the SEs. Means with different letters are statistically different (*p* = 0.05; *n* = 4) as determined by LSD multiple comparison test.

Figure 6 shows F_v_/F_m_ ratio, the light-response curves of ETR, ΔF*/*F_m_', and NPQ after *M. crystallinum* transferred from full N to different N treatments for 7 days. All plants had F_v_/F_m_ ratios higher than 0.8 (Figure 6A). ETR (Figure 6B) and NPQ (Figure 6D) increased, while ΔF/F_m_' (Figure 6C) decreased with increasing PPFD for all plants. The light-response curves of ETR and ΔF/F_m_' for 0 N plants were above those of other plants. However, the light-response curves of NPQ of *M. crystallinum* grown with full N for the whole treatment period were above those transferred from full N to other reduced N treatments. When measured under the actinic light near the growth light at a PPFD of 231 μmol m^−2^ s^−1^, there were no significant differences in ETR (Figure 6B) and ΔF/F_m_' (Figure 6C) among the different treatments. However, under this actinic light, *M. crystallinum* grown with full N had significantly higher NPQ compared with all other plants (Figure 6D). Compared with other plants, 0 N plants had significantly higher ETR and ΔF/F_m_', while NPQ was significantly higher in full N plants when measurements were made under the highest actinic light at a PPFD of 1,076 μmol m^−2^ s^−1^ (Figures 6B–D).

### Proline, TSS, ASC, and Total Phenolic Compounds

**Figure 7:**
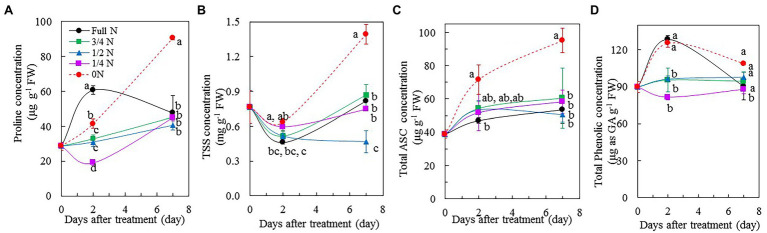
Changes of proline **(A)**, total soluble sugars (TSS; **B**), total ascorbic acid (ASC; **C**), and total phenolic compounds **(D)** concentration in the leaves of *M. crystallinum* after different NO_3_^–^ treatments. Vertical bars represent the SEs. Means with different letters are statistically different (*p* = 0.05; *n* = 4 for **A,C,D**; *n* = 5 for **B**) as determined by LSD multiple comparison test.

*Mesembryanthemum crystallinum* plants transferred from full N to 0 N showed increases in proline (Figure 7A), TSS (Figure 7B), and ASC concentrations (Figure 7C). The concentrations of proline, TSS, and ASC were significantly higher in 0 N plants compared with all other plants, which had similar levels of these three parameters during the treatment. After transferring them from full N to 0 N for 7 days, the concentrations of proline, TSS, and ASC were double compared to the rest of plants, although there were some variations of these three parameters among the different treatment after 2 days. For the total phenolic compounds, full N and 0 N plants had significantly higher concentration compared to other plants after 2 days of treatment. However, all plants had similar level of total phenolic compounds except for those transferred from full N to 1/4 N conditions for 7 days (Figure 7D).

## Discussion

### Productivity of Shoot and Root and Leaf Growth

Being required in the largest amount among all essential elements for plant growth and development, N is a major factor limiting plant growth in natural (Marschner, 1995) as well as agricultural (Bian et al., 2020) environments. NO_3_^–^ is mainly absorbed by plant roots from the growth media, such as soil or nutrient solution. For soilless culture, NO_3_^–^ fertilizer is mainly used to ensure high yield (Marschner, 1995). Insufficient supply or oversupply of NO_3_^–^ could affect both productivity and quality (Bian et al., 2020). A nutritious succulent halophyte, *M. crystallinum*, is getting its popularity as a read-to-eat salad in Singapore. We have successfully cultivated them indoor with both fresh and saline water nutrient solution under LED lighting. We have also found that all *M. crystallinum* plants had adequate levels of N in their tissues regardless of growth conditions, optimal or suboptimal (He et al., 2017, 2020; He and Qin, 2020). However, the effects of NO_3_^–^ supply on the growth, NO_3_^–^ content, and nutritional quality of *M. crystallinum* are lacking.

NO_3_^–^ concentration in leaf vegetables can be manipulated by reducing or withdrawing NO_3_^–^ supply prior to harvesting (He et al., 2011a, 2014; Conversa et al., 2021). In this study, all *M. crystallinum* plants were first grown with full N for 11 days before transferring them to 3/4 N, 1/2 N, 1/4 N, and 0 N conditions for another 7 days. The total growing period was 18 days. Although all plants were healthy and grew well (Figure 1A), it was surprising to note that *M. crystallinum* plants that continued to grow with full N were smaller (Figures 1A–C) compared with those transferred to 3/4 N and 1/2 N conditions for 7 days. These results indicate that *M. crystallinum* grown hydroponically under a low level of LED lighting, supplied with a full-strength Netherlands Standard Composition solution containing N-NO_3_^–^ (13.37 mM), may have been subjected to stress resulting from excessive N. The negative impact of excessive N on plant growth and biomass accumulation was mainly due to the reduction of photosynthetic performance (Nakaji et al., 2002; Jauffrais et al., 2016). However, there are limited studies on the effects of excessive N on growth and biomass accumulation of vegetable crops. In this study, it was found that plants which transferred to 1/4 N had similar shoot FW and root FW as those grown with full N. It is no doubt that the lowest values of shoot and root FW obtained from *M. crystallinum* transferred from full N to 0 N resulted from N deficiency. We have previously reported that when butter head lettuce (*L. sativa*) were grown under full sunlight, plants supplied with full N had the highest shoot FW followed by those with 1/2 NO_3_^–^, and the lowest shoot FW was recorded in plants grown without NO_3_^–^ for 7 days (He et al., 2011a). He et al. (2014) found that shoot and root productivities of two lettuce RILs grown under high N (+N, 125% NO_3_^–^) and low N (–N, 50% NO_3_^–^) declined compared to those grown under full N (100% NO_3_^–^). N deficiency stunts plant growth and development and ultimately decreases plant productivity, which have also been reported by others in lettuce (Broadley et al., 2001). N deficiency significantly reduced photosynthetic capacity, resulting in lower biomass accumulation (He et al., 2011a, 2014; Mu and Chen, 2021).

In this study, *M. crystallinum* plants which were transferred from full N to 0 N had significantly higher shoot/root FW ratio than other plants (Figure 1D). Most plants would change their shoot/root ratios in response to nutrient availability (Tolley and Mohammadi, 2020). As NO_3_^–^ is not only a major N source of nutrition for plants but also acts as a signal to modulate root development, Crawford and Glass (1998) hypothesized that root cells have a sensor for NO_3_^–^ availability. For example, the initiation and elongation of *Arabidopsis* lateral root development was stimulated by local availability of NO_3_^–^ (Little et al., 2005). In high-N environments, increasing root biomass was associated with greater N uptake and higher yield (Gastal et al., 2015; Tolley and Mohammadi, 2020). On the other hand, N deficit increases the root/shoot ratio or decreases the shoot/root ratio (Gastal et al., 2015). However, variation exists between species in the intensity of the shoot/root response to N deficiency (Azimi et al., 2021). This explains the results of *M. crystallinum* plants transferred from full N to 0 N having higher shoot/root FW ratio, which was different from those reported by others (Gastal et al., 2015; Tolley and Mohammadi, 2020).

Shoot biomass accumulation was mainly due to increases in leaf number and leaf area. Leaf area determines light interception and is an important parameter in determining plant productivity (He et al., 2019). The reductions of leaf number (Figure 2A) and TLA (Figure 2B) might partly account for the lower biomass for *M. crystallinum* grown with full N and for those transferred from full N to 1/4 N and 0 N. N deficiency significantly reduced leaf area, resulting in lower photosynthetic capacity leading to lower biomass production (Broadley et al., 2001; Tolley and Mohammadi, 2020; Azimi et al., 2021; Mu and Chen, 2021). However, there is very little information on the effects of excessive N on leaf number and TLA. SLA, which is a measure of thickness of the leaf, is a key trait explaining growth variations of plant species under different environments. N supply is an important factor that determines SLA (Koch et al., 1988; de Ávila Silva et al., 2021). It was reported that N-deficient wild radish plants had higher SLA compared with those grown under N-sufficient conditions (Koch et al., 1988). In the study with pepper (*Capsicum annuum*), de Ávila Silva et al. (2021) found that SLA decreased in response to increased N supply. In this study, compared with *M. crystallinum* grown with full N, 0 N plants had significantly lower SLA. However, compared with plants transferred to 1/2 N, SLA was lower in *M. crystallinum* grown with full N (Figure 2C). These results suggest that both N-deficient and N-excessive conditions could result in decreases in SLA of *M. crystallinum* in the present study.

### Leaf Water Status

*Mesembryanthemum crystallinum* plants transferred from full N to 0 N, which resulted in a reduction of leaf expansion within 2 days (Figure 2B). Palmer et al. (1996) reported that a reduction in N supply reduced leaf expansion of young sunflower (*Helianthus annuus* L.) within 24 h. N deprivation may reduce root cell hydraulic conductivity and the overall plant hydraulic conductivity, thus leading to the decrease of leaf turgor. In this study, although the LWC of 0 N plants was not significantly different from those of full N plants after 7 days of treatment, it was the lowest among all plants (Figure 3C). The consequent decline in LWC may lead to a decline in leaf cell turgor for the 0 N plants. Furthermore, the different responses to different NO_3_^–^ availability in SLA (Figure 2C) may result from the differences not only in TLA (Figure 2B) but also in LWC (Figure 3C) and LDMC (Figure 3B). LDMC (mg g^−1^) has been proposed as an indicator of plant resource use such as N (Garnier et al., 2001). It is the proportion of the leaf matter content without water related to the mass of the leaf with the maximum water content. In this study, LDMC was higher in 0 N plants than in other plants, indicating that 0 N plants accumulated more biomass for the same amount of FW. However, in the study with maize (*Zea mays* L.; Kulsum et al., 2007) and wheat (*Triticum aestivum* L.; Tolley and Mohammadi, 2020), it was found that with the increasing N levels, dry leaf mass increased. Response of LDMC to reduced N may vary among different species as well as other growth conditions. In our recent study, it was found that high salinity resulted in lower LS in *M. crystallinum* (He et al., 2021a). In this study, the LS of *M. crystallinum* plants was not affected by the availability of NO_3_^–^ when they were grown with 10% ASW (Figure 3A).

### Shoot NO_3_^–^ Concentrations, NRA, and Leaf Total Soluble Protein

Reduced NO_3_^–^ availability in the growth media results in reduced accumulation of NO_3_^–^ in edible portions of crop plants (He et al., 2014; Bian et al., 2020; Conversa et al., 2021). However, in the present study, compared with *M. crystallinum* plants grown with full N, those transferred from full N to 3/4 N, 1/2 N, and 1/4 N seemed to be constant or slightly increased in shoot NO_3_^–^ concentrations during the reduced NO_3_^–^ treatments on a DW (Figure 4A) or a FW basis (Figure 4B). Shoot NO_3_^–^ concentration decreased in *M. crystallinum* plants transferred from full N to 0 N during 7-day treatment compared with that of full N plants (Figures 4A,B). Recently, Conversa et al. (2021) reported that the NO_3_^–^ level decreased after 3 days of treatment for baby-leaf lettuce (*L. sativa* var. *longifolia* L.) or 6 days of treatment for *Cichorium endivia* L. after nutrient solution withdrawal. In this study, based on the results shown in Figure 4B, the shoot NO_3_^–^ concentrations of *M. crystallinum* grown with full N, 3/4 N, 1/2 N, 1/4 N, and 0 N after 7 days of different NO_3_^–^ treatments were 763, 1,315, 871, 941, and 416 mg kg ^−1^ FW, respectively. Each of the NO_3_^–^ concentrations was below the limits set for other leafy vegetables grown in the greenhouse (Zhong et al., 2002). Although N is a primary macronutrient required in the greatest amount for plant growth and development, plants are no longer able to uptake NO_3_^–^ when supply of it exceeds the demand, and NO_3_^–^ may undergo efflux to the apoplast and then the surrounding environment (Nosengo, 2003). According to McCall and Willumsen (1998), high rates of NO_3_^–^ application increased the plant NO_3_^–^ content without increasing the yield. However, based on the results of plant growth in this study (Figures 1, 2), *M. crystallinum* grown with full N is very likely to be exposed to excessive N, which resulted in reduced biomass accumulation (Nakaji et al., 2002).

In this study, it was also noticed that *M. crystallinum* plants transferred from full N to 3/4 N, 1/2 N, and 1/4 N seemed to have constant or slightly increased shoot NO_3_^–^ concentrations compared with those of full N plants during the treatments (Figures 4A,B). Higher plants acquire NO_3_^–^ from their surrounding environment through the activities of both high-affinity transport systems (HATS) and low-affinity transport systems (LATS), respectively. It is assumed that root NO_3_^–^ uptake is mostly determined by the activity of the HATS (Malagoli et al., 2004; Anthony et al., 2007). In a soilless culture system, as plants are supplied with nutrient solution continuously, increases in NO_3_^–^ uptake and transport could result from activities of both HATS and LATS (Malagoli et al., 2004). It is possible that when *M. crystallinum* transferred from full N to 3/4 N, 1/2 N, and 1/4 N, reduced NO_3_^–^ supply activated HATS, which functioned as a sensor for root NO_3_^–^ availability and thus increased NO_3_^–^ uptake and transport (Anthony et al., 2007), especially for the 3/4 N plants. Gradual decrease in shoot NO_3_^–^ concentration after transferring *M. crystallinum* from full N to 0 N conditions was expected to be seen.

Shoot NO_3_^–^ accumulation depends not only on its net absorption but also on its assimilation rate. The amount of NO_3_^–^ accumulated in the plant tissues is closely related to NRA (Bian et al., 2020). In a study with the native Australian plant Boronia (*Boronia megastigma* Nees), Reddy and Menary (1990) reported that NRA was affected by NO_3_^–^ availability and plants could not reduce NO_3_^–^ at high rates when NO_3_^–^ supply was high, leading to high accumulation of NO_3_^–^. In the study with lettuce RILs, He et al. (2015) reported that shoot NO_3_^–^ concentration and maximal NRA were higher in plants supplied with high NO_3_^–^ compared with those supplied with lower NO_3_^–^. In this study, a similar correlation between shoot NO_3_^–^ concentration and maximal NRA was also observed after 7 days of different NO_3_^–^ treatments (Figures 4A–C). C_3_ leaves typically invested 20–30% of leaf N to soluble protein (Sage et al., 1987). Low N supply may alter the allocation of leaf N to soluble proteins (Mu et al., 2016). However, in this study, *M. crystallinum* plants which transferred from full N to 0 N had the highest total leaf soluble protein compared with other treatments (Figure 4D). This finding could be due to the protein that was concentrated within a small plant grown under 0 N condition.

### Chl, F_v_/F_m_ Ratio, E.TR, ΔF*/*F_m_', and NPQ

N is a key element for the biosynthesis of Chl in plants (Sage et al., 1987; Mu and Chen, 2021). Sage et al. (1987) reported that leaf Chl content was positively correlated with N. Under low N stress, Chl content decreased in both C_3_ plants, such as rice (*Oryza sativa* L.), wheat (*T. aestivum* L.), soybean (*Glycine max* L.), and *Populus cathayana*, and C_4_ plants, such as maize (*Z. mays* L.), C4 grasses, and sorghum [*Sorghum bicolor* (L) Moench] (Mu and Chen, 2021). In this study, there were no clear trends in Chl a, Chl b, and total Chl concentrations in *M. crystallinum* among the different NO_3_^–^ treatments (Figures 5A–C). According to our observation, compared with all other plants, 0 N plants appeared greener during the 7-day treatment period (Figure 1A), which was supported by their higher Chl concentrations (Figures 5A–C). Higher Chl concentration in 0 N plants could be due to their pigments concentrated with small leaves. According to Mu and Chen (2021), different plants have different responses for Chl allocation under N stress. It is also possible for *M. crystallinum* to invest more N to Chl in an N-deficient environment. All *M. crystallinum* plants had similar Chl a/b ratio before and during the 7-day treatment period (Figure 5D). In their review paper, Mu and Chen (2021) summarized that the total Chl decreased as N supply decreased, while the Chl a/b ratio remained constant in rice (*O. sativa* L.), soybean (*G. max* L), and *P. cathayana*.

Nitrogen deficiency reduced photosynthetic capacity, leading to lower biomass accumulation (Zhao et al., 2005; Mu and Chen, 2021). It was also reported that excessive N inhibited photosynthesis and productivity (Nakaji et al., 2002; Cun et al., 2021). This study focused on the impacts of NO_3_^–^ supply on photosynthetic light-use efficiency. The maximum quantum yield of PSII measured by Chl fluorescence F_v_/F_m_ ratio from the dark-adapted leaves is an early indicator of plants response to stress (Matsuoka et al., 2018). After 7 days of different NO_3_^–^ treatments, all *M. crystallinum* plants had their F_v_/F_m_ ratios greater than 0.8 (Figure 6A), indicating that the maximum quantum yield of PSII was not affected by NO_3_^–^ availability. It is interesting to see that ETR and effective quantum yield of PSII measured by ΔF/F_m_' were higher in 0 N plants than in all other plants under high actinic light (Figures 6B,C). However, NPQ of *M. crystallinum* grown with full N was higher compared with other plants transferred from full N to reduced N conditions (Figure 6D). These results contradict the findings of others who reported that N deficiency decreased F_v_/F_m_ ratios, ETR, and ΔF/F_m_' but increased NPQ (Mu et al., 2017). Higher ETR and ΔF/F_m_', especially measured under high actinic light in 0 N plants (Figures 6B,C), could be due to its higher Chl content (Figures 5A–C) and thus higher photon absorption. Cun et al. (2021) reported that F_v_/F_m_ ratio, ΔF/F_m_', and NPQ decreased in N-sensitive plant species grown with high N compared with those grown with moderate N. However, in this study, NPQ was significantly higher in full N plants than in other plants when the measurements were made under both the actinic light near the growth light and the highest PPFD of 1,076 μmol m^−2^ s^−1^ (Figure 6D). This result suggests that *M. crystallinum* may experience excessive N supply.

### Proline, TSS, ASC, and Total Phenolic Compounds

Proline is a metabolite with multiple roles, such as antioxidant and osmoprotectant, in plants (Szabados and Savouré, 2010). Proline accumulates in plants when exposure to different types of environmental stresses, including drought, salinity, and nutrient deficiency (Hayat et al., 2012). In this study, after transferring *M. crystallinum* from full N to 0 N for 7 days, the concentration of proline was double compared with the rest of plants (Figure 7A). The increase of proline could serve as an indicator for unbalanced N nutrition (Atanasova, 2008). In the study with maize seedlings, Göring and Thien (1979) reported that proline accumulation was induced by nutrient deficiency. Apart from proline, TSS and ASC concentrations of 0 N plants also increased significantly, and they were also double compared with those of other plants after 7 days of treatment (Figures 7B,C). According to Blom-Zandstra and Lampe (1983), NO_3_^–^ accumulation negatively correlated with sugar and dry matter accumulation in different lettuce genotypes to maintain their osmotic value (Reinink et al., 1987). In the study with soybean plants [*G. max* (L.) Merrill, “Ransom”], Rufty et al. (1988) reported that N deficiency led to high carbohydrate accumulation in plants, such as starch and sucrose. Khan et al. (2018) found that a gradual decrease in NO_3_^–^ content was accompanied by an increased concentration of nutritional carbohydrates in three lettuce plants after increasing light intensity and lowering NO_3_^–^ supply prior to harvest. All these earlier findings are in line with the results of this study. The significant increases of ASC during the 0 N treatment period in the present study might be ascribed to the accumulation of carbohydrate, which is an important substrate for ASC biosynthesis, induced by N deficiency. This was supported by the study with cucumber leaves (Zhang et al., 2016). The bladder cells on the surfaces of leaf and stem of *M. crystallinum* contain high concentrations of useful phytochemicals, including phenolic compounds which could be manipulated by light quality (Kim et al., 2021), drought (He et al., 2020), and salt stress (He et al., 2021a). However, in this study, reduced NO_3_^–^ supply did not result in significant changes of total phenolic compounds except for those plants which were transferred from full N to 1/4 N conditions (Figure 7D).

## Conclusion

To the best of our knowledge, this was the first project to study the impacts of reduced NO_3_^–^ supply on productivity, N metabolism, photosynthetic light-use efficiency, and nutritional values in edible *M. crystallinum* grown indoor. All *M. crystallinum* plants grew well and healthy after transferring them from full N to different reduced NO_3_^–^ nutrient solutions for 7 days. When considering productivity, based on this study, full N supply for the whole growth cycle (18 days) is not necessary to achieve the highest shoot FW. Instead, *M. crystallinum* plants grown with full N had lower biomass accumulation compared with those supplied with reduced NO_3_^–^ to 75 (3/4 N) or 50% (1/2 N). Shoot NO_3_^–^ accumulation in all plants was below the harmful limits set for other leafy vegetables grown in the greenhouse with the lowest values found in *M. crystallinum* plants transferred from full N to 0 N. Shoot NO_3_^–^ accumulation was associated with NRA. Although *M. crystallinum* plants transferred from full N to 0 N had the lowest shoot FW, they had higher Chl content coupled with higher ETR and quantum yield of PSII measured by ΔF/F_m_' than other plants. The 0 N plants also had much higher concentrations of proline, TSS, and total ASC than other plants. A reduction in NO_3_^–^ accumulation could add value to *M. crystallinum*, which is already very popular for its nutritional properties. Therefore, it is important to adopt appropriate strategies to optimize the amount of NO_3_^–^ used in soilless culture media for *M. crystallinum* in order to further enhance its nutritional values. Based on the results of this study, totally withdrawing NO_3_^–^ prior to harvest could be one of the strategies as the concentrations of proline, TSS, and ASC were enhanced markedly in *M. crystallinum*.

## Data Availability Statement

The original contributions presented in the study are included in the article/Supplementary Material, further inquiries can be directed to the corresponding author.

## Author Contributions

JH initiated and funded the expenses for the project and wrote the first draft of the manuscript. JH and LQ planned the experiments, carried out the experiments, analyzed the data, and revised the manuscript. LQ plotted the graphs. All authors contributed to the article and approved the submitted version.

### Conflict of Interest

The authors declare that the research was conducted in the absence of any commercial or financial relationships that could be construed as a potential conflict of interest.
