# Supplementary figures and images for: Impacts of Reduced Nitrate Supply on Nitrogen Metabolism, Photosynthetic Light-Use Efficiency, and Nutritional Values of Edible Mesembryanthemum crystallinum
Source: Front Plant Sci. 2021 Jun 4;12:686910. doi: 10.3389/fpls.2021.686910 (PMC8213338; doi:10.3389/fpls.2021.686910)

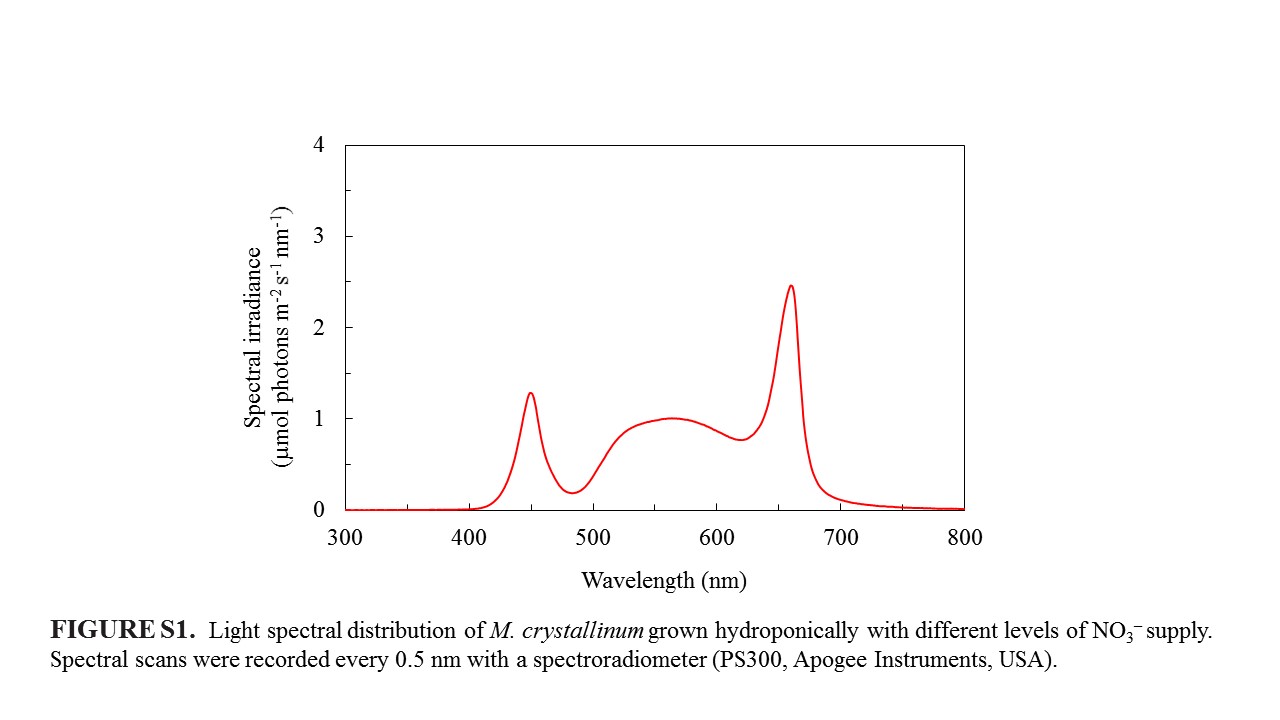

Supplement: Supplementary file 1 [file Image_1.JPEG]
